# Supplementary material for: The causal relationship between the human gut microbiota and pyogenic arthritis: a Mendelian randomization study
Source: Front Cell Infect Microbiol. 2024 Nov 26;14:1452480. doi: 10.3389/fcimb.2024.1452480 (PMC11629706; doi:10.3389/fcimb.2024.1452480)
Supplement: Supplementary file 1 [file DataSheet1.zip › mendelian test/ebi-a-GCST90027723.csv_scatter.pdf]

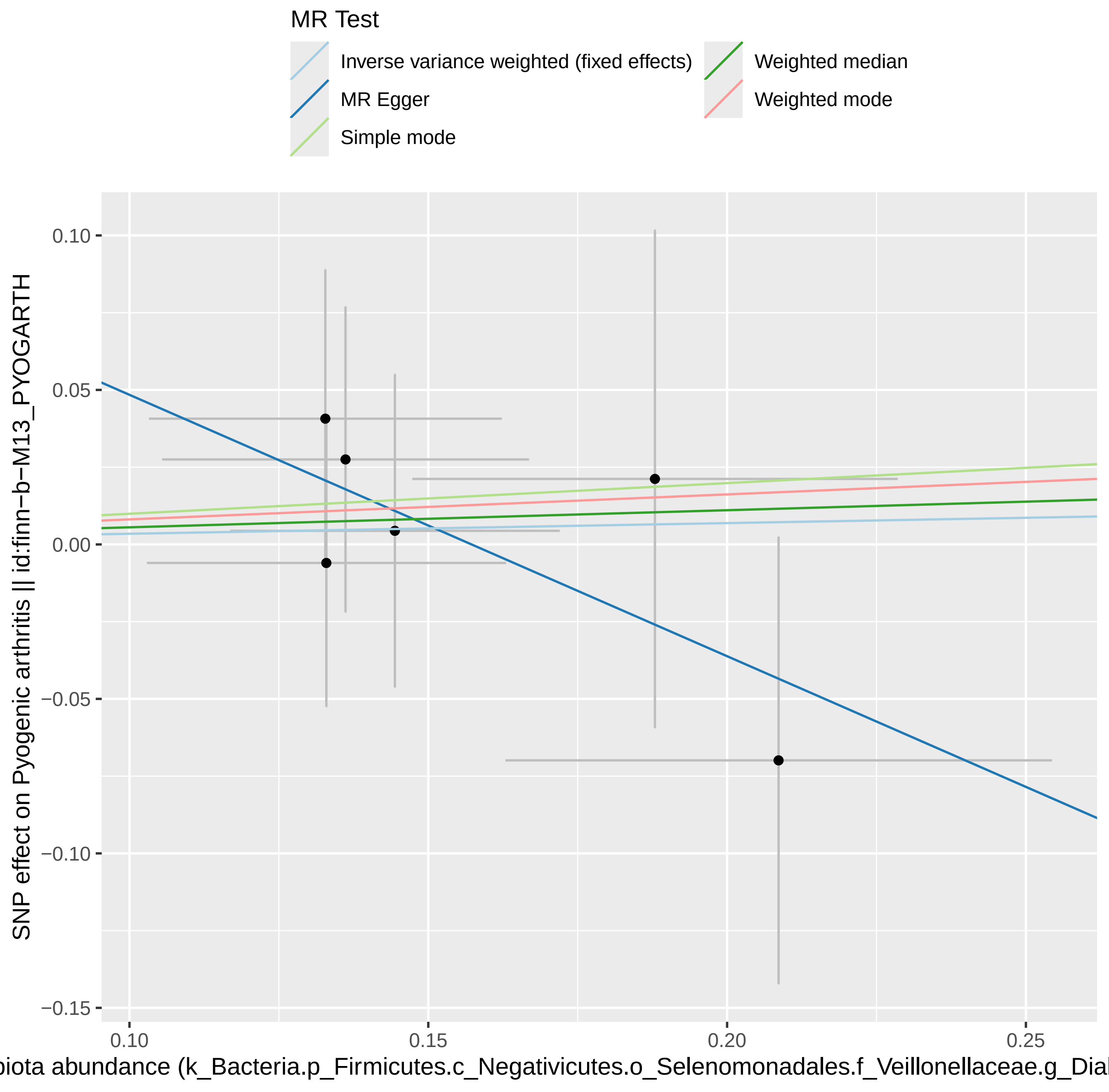
MR Test
Inverse variance weighted (fixed effects)
MR Egger
Simple mode
Weighted median
Weighted mode
SNP effect on Pyogenic arthritis || id:finn−b−M13_PYOGARTH
0.10
0.05
0.00
−0.05
−0.10
−0.15
0.10
0.15
0.20
0.25
biota abundance (k_Bacteria.p_Firmicutes.c_Negativicutes.o_Selenomonadales.f_Veillonellaceae.g_Dial
